# Glibenclamide Advantage in Treating Edema After Intracerebral Hemorrhage (GATE-ICH): Study Protocol for a Multicenter Randomized, Controlled, Assessor-Blinded Trial

**DOI:** 10.3389/fneur.2021.656520

**Published:** 2021-04-27

**Authors:** Jingjing Zhao, Fang Yang, Changgeng Song, Li Li, Xiai Yang, Xiaofeng Wang, Liping Yu, Jun Guo, Kangjun Wang, Feng Fu, Wen Jiang

**Affiliations:** ^1^Department of Neurology, Xijing Hospital, Fourth Military Medical University (Air Force Medical University), Xi'an, China; ^2^Department of Neurology, Ankang Central Hospital, Ankang, China; ^3^Department of Neurosurgery, The PLA 987 Hospital, Baoji, China; ^4^Department of Neurology, The First Peoples Hospital of Xianyang, Xianyang, China; ^5^Department of Neurology, Tangdu Hospital, Fourth Military Medical University (Air Force Medical University), Xi'an, China; ^6^Department of Neurology, Hanzhong Central Hospital, Hanzhong, China; ^7^Department of Neurology, 215 Hospital of Shaanxi NI, Xianyang, China

**Keywords:** perihematomal edema, intracerebral hemorrhage, glibenclamide, prognosis, clinical trial

## Abstract

**Introduction:** Brain edema after acute intracerebral hemorrhage (ICH) plays a critical role in the secondary injury of ICH and may heighten the potential for a poor outcome. This trial aims to explore the efficacy of small doses of oral glibenclamide in perihematomal edema (PHE) and the prognosis of patients with ICH.

**Methods and Analysis:** The GATE-ICH trial is a multicenter randomized, controlled, assessor-blinded trial. A total of 220 adult patients with acute primary ICH in 28 study centers in China will be randomized to the glibenclamide group (glibenclamide plus guideline-recommended ICH management) or the control group (guideline-recommended ICH management). Multivariate logistic regression will be used to analyze the relationship between the treatments and primary outcome.

**Study Outcomes:** The primary efficacy outcome is the proportion of poor functional outcomes (modified Rankin Scale ≥3) at 90 days after enrollment. The secondary efficacy outcomes include changes in the volume of ICH and PHE between the baseline and follow-up computed tomography scans as well as the clinical scores between the baseline and follow-up assessments.

**Discussion:** The GATE-ICH trial will assess the effects of small doses of oral glibenclamide in reducing the PHE after ICH and improving the 90-day prognosis of patients.

**Clinical Trial Registration:** www.clinicaltrials.gov., NCT03741530. Registered on November 8, 2018.

**Trial Status:** Protocol version: May 6, 2019, Version 5. Recruitment and follow-up of patients is currently ongoing. This trial will be end in the second quarter of 2021.

## Introduction

Intracerebral hemorrhage (ICH) accounts for 10–15% of the 15 million strokes worldwide each year and has a high mortality and morbidity (1). Compared with Western populations, the incidence of ICH in the Chinese population is higher (1, 2). However, only 12–39% of ICH survivors achieve long-term functional independence (3, 4). Despite the severe outcomes, there are no effective medical or surgical therapeutics available (5).

Perihematomal edema (PHE) after primary ICH, as a combination of early vasogenic edema following cytotoxic edema (6, 7). It is the most critical factor of secondary brain injury that leads to additional neurological deterioration (8, 9). The volume of PHE increases by ~75% during the first 24 h after ICH. It peaks in ~5–6 days, and continues for 14 days (1). Managing PHE may be an optimal target for ameliorating secondary brain injury in patients with ICH (10).

Most recently, glibenclamide, a sulfonylurea (SFU) drug and potent inhibitor of the sulfonylurea receptor 1-transient receptor potential melastatin 4 (SUR1-TRPM4) channels, has attracted great interest as a promising therapy for the prevention of cerebral edema after hemispheric infarction. It has been verified in the GAMES-RP study (11–13). Glibenclamide may contribute to mitigating both cytotoxic and vasogenic edema by blocking the SUR1-TRPM4 channels, inhibiting apoptosis, scavenging free radicals, and protecting the integrity of the blood-brain barrier (BBB) (14–16). In patients with ICH with a history of diabetes, SFU used prior to ICH could reduce the accumulation of ICH and PHE volume and improve the short-term prognosis (17, 18). Given the safety and efficacy of oral glibenclamide in treating edema after brain injury, we supposed that the oral formulation of glibenclamide would be efficacious in preventing PHE in patients with acute ICH.

## Methods and Analysis

### Study Objectives

The purpose of this hospital-based study is to assess whether small doses of oral glibenclamide would reduce edema and improve the prognosis in patients with ICH.

### Study Design

The Glibenclamide Advantage in Treating Edema after Intracerebral Hemorrhage (GATE-ICH) study is a multicenter, randomized, controlled, assessor-blinded clinical trial. The subjects will be randomized into one of the two intervention arms using a web-based 1:1 centralized randomization process (http://cerebralhemorrhage.applinzi.com. computerized random numbers): (1) the glibenclamide group (glibenclamide plus guideline-recommended ICH management), and (2) the control group (guideline-recommended ICH management). There are four time points of assessment: baseline (t0), 3 days (t1), 7 days (t2), and 90 days (t3) after enrollment (Table 1). The Supplementary File 1 describes the Recommendations of Interventional Trials (SPIRIT), which indicates the recommended items and corresponding page numbers of the present protocol.

**Table 1:** Overview of assessment points and measurements.

**Assessment points**	**Time range**
t0	baseline assessment prior to enrollment
t1	3 days (72 h ± 12 h) post-enrollment
t2	7 days (168 h ± 12 h) post-enrollment
t3	90 days (± 7 d) post-enrollment

### Study Population

The GATE-ICH trial (www.clinicaltrials.gov. NCT03741530) involves 26 hospitals in the Shaanxi Province of China. We will conduct the study based on the recommendations of the Good Clinical Practice guidelines and the Declaration of Helsinki. The trial has been approved by the ethics committee of Xijing Hospital (KY20182067-X-3). The study will include patients with acute primary ICH who have ganglia hemorrhage of 5–30 mL confirmed by a baseline head computed tomography (CT). The inclusion criteria and exclusion criteria are shown in Box 1. Potential patients will be identified by emergency physicians and the initial neurologists. Trained neurologists at every center will be requested to make a definite diagnosis of ICH and confirm that each patient has met the inclusion criteria of the study. A written informed consent form of the present study and additional *post-hoc* analysis will be sent to all of the subjects or their guardians by the trained neurologists.

Box 1 **Inclusion and exclusion criteria.**

**Inclusion criteria**

1. Age 18–70 years with primary ICH.
2. Baseline CT with basal ganglia hemorrhage of 5–30 mL.
3. GCS score ≥ 6.
4. Symptom onset <72 h prior to admission.
5. Informed consent.

**Exclusion criteria**

1. Supratentorial ICH planned to evacuation of a large hematoma.
2. Hemorrhage breaking into ventricles of the brain.
3. Prior significant disability (mRS ≥ 3).
4. Severe renal disease (i.e., renal disorder requiring dialysis) or eGFR <30 ml/min/1.73 m^2^.
5. Severe liver disorder, or ALT >3 times or bilirubin >2 times upper limit of normal.
6. BG <3.1 mmol/L at enrollment, or with the history of hypoglycemia.
7. Having acute ST elevation infarction, or decompensated heart failure, or cardiac arrest, or acute coronary syndrome, or known history of acute coronary syndrome, or acute myocardial infarction, or coronary intervention in the past 3 months.
8. Treatment with sulfonylurea in the past 7 days, including glyburide, glyburide plus metformin, glimepiride, repaglinide, glipizide, gliclazide, tolbutamide, and/or glibornuride.
9. Treatment with bosentan in the past 7 days.
10. Having an allergy to sulfa or other sulfonylurea drugs.
11. Known G6PD deficiency.
12. Pregnant women.
13. Breast-feeding women disagreeing to participate the study or to discontinue breastfeeding during and after the study.
14. Being enrolled in another study and receiving an investigational drug.
15. Refusing to be enrolled, having poor compliance, and/or tending to withdraw.

ALT, Alanine aminotransferase; BG, Blood glucose; CT, computed tomography; eGFR, estimated glomerular filtration rate; GCS, Glasgow Coma Scale; G6PD, Glucose-6-phosphate Dehydrogenase; ICH, intracerebral hemorrhage; mRS, modified Rankin Scale.

### Randomization

The baseline data of potential participants will be provided to the research coordinators by investigators at every center. Through a web-based 1:1 randomization (computerized random numbers) process (http://cerebralhemorrhage.applinzi.com/index.php), patients will be assigned to one of the two intervention arms by the research coordinators.

### Training of Investigators

To ensure the quality of the GATE-ICH trial, all the investigators from every center are required to receive official training regarding the study protocol, Good Clinical Practice guidelines, clinical scores including the Glasgow Coma Scale (GCS), National Institutes of Health Stroke Scale (NIHSS), ICH score, Barthel index, and modified Rankin Scale (mRS), as well as the recognition of basal ganglia hemorrhage on head CT.

### Trial Interventions

#### Intervention Arms

The time course of plasma glibenclamide levels indicates that an oral formulation of 3.5 mg/day at 4 h after administration is equal to an intravenous formulation of a 0.13-mg bolus followed by a dose of 3 mg/day, which has been proved efficacious and safe for treating edema in patients with ischemic stroke (13, 19). The half-life of glibenclamide is 4.0–13.4 h in elderly patients and 4.0–13.9 h in younger patients (20). In order to achieve a stable drug concentration that coincides with three mealtimes, we chose a dose of 1.25 mg, 3 times per day (3.75 mg/day). In our pilot study, 10 patients in the glibenclamide group and 12 patients in the control group were enrolled. The plasma concentration of glibenclamide elevated to 47.6 ng/mL at 2 h after the first dose, and gradually achieved by steady state plasma concentration of glibenclamide at 72 h (26.7 ng/mL). Patients assigned to the glibenclamide group will receive tablets containing 1.25 mg of glibenclamide [Yun Peng Pharmaceutical Co., Shanxi, China; each capsule (2.5 mg)] three times daily (no more than 30 min before a meal) for 7 consecutive days after enrollment in addition to the usual care and medications for ICH. Patients in the control group will receive only the usual care and medications for ICH. Glibenclamide tablets should not be administered after head CT on day 7. Treatment with other SFU agents is not permitted during the 90-day follow-up period in either group.

#### Patient Safety

The study drug must be reduced to 1.25 mg twice daily if any of the following conditions are present: (1) laboratory-confirmed blood glucose (BG) level <3.1 mmol/L; (2) or three laboratory-confirmed BG levels <3.9 mmol/L within 12 h. The study drug must be discontinued if either of these conditions occur twice. In patients with hyperglycemia during their hospitalization, increasing the dosage of glibenclamide or substituting glibenclamide with other SFU agents is not permitted. In patients with a laboratory-confirmed BG level <3.9 mmol/L, administration of 50% glucose is suggested, according to the following formula: supplementation volume of 50% glucose = [100–laboratory-confirmed BG (mg/dL)] × 0.4 mL. Other highly hyperosmotic glucose solutions with the same amount of sugar are also allowed.

#### Medical Care in the Hospital

Both groups in the present study will receive the same background care. Traditional dehydration therapy is not recommended for patients without intracranial hypertension. In patients with intracranial hypertension, the selection of osmotherapy medicines depends on local availability. The use of supplemental fluids is based on the clinical status of the subjects. In patients randomized to the glibenclamide group with inadequate intake, but not meeting the standard nasal feeding, 5 or 10% glucose is recommended to avoid hypoglycemia. The management of blood pressure and all other medical care for ICH patients will adhere to the ICH guidelines (21).

### Study Procedures

**Figure 1:**
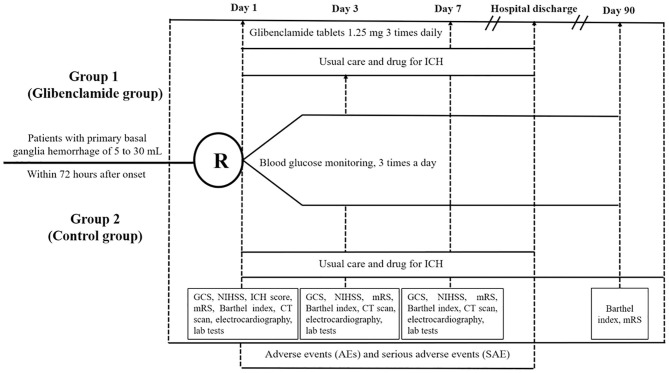
Flowchart of the trial design.

Figure 1 presents the flow chart of the GATE-ICH trial. Based on the inclusion and exclusion criteria, the potential subjects offering informed consent will be randomized. At baseline, demographics, medical history (acute ischemic stroke, ICH, coronary events, diabetes mellitus, and hypertension), physical examination results, clinical scores (NIHSS, GCS, ICH score, Barthel index, mRS), vital signs, and head CT results will be recorded. Routine laboratory tests for patients with ICH will be conducted (routine blood tests, renal and liver function tests, serum lipid, fasting glucose, routine urine tests, electrocardiography, and head CT) on days 1, 3, and 7. Clinical scores (NIHSS, GCS, Barthel index, and mRS) on day 3 and day 7 or the day of hospital discharge will be collected. The Barthel index and mRS on day 90 will be assessed. During the entire period of hospitalization, concomitant treatments, adverse events (AEs), and serious AEs (SAEs) will be documented.

#### Imaging Evaluations

**Figure 2:**
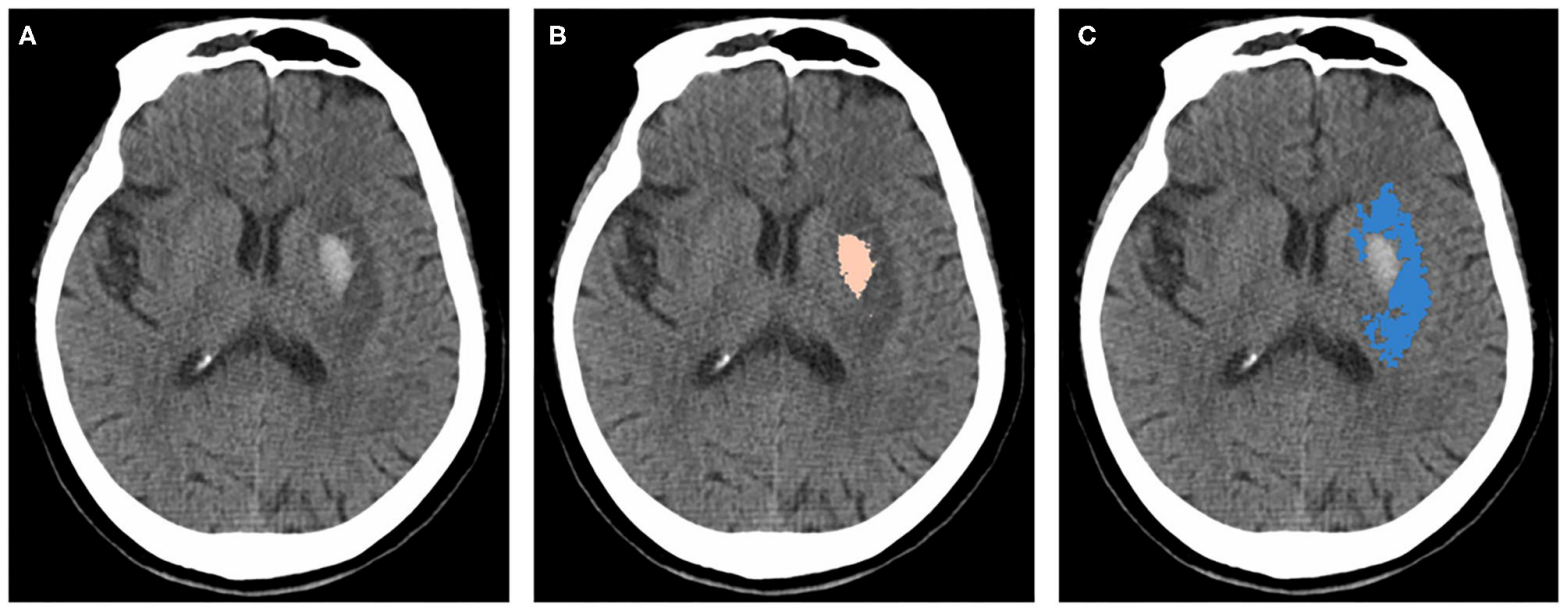
Measurements of hematoma and PHE using 3D slicer software package. **(A)** original image of a patient with ICH. **(B)** hematoma marked with pink. **(C)** PHE marked with blue. ICH, intracerebral hemorrhage; PHE, perihematomal edema.

A baseline CT evaluation of ICH volume will be performed by the investigators and study coordinators in each center using an image matrix of 512 × 512 with a slice thickness of 5 mm. The three CT assessments of the same subject should use the same type of CT. At an initial meeting, all investigators will be trained on the methodology of hematoma volume calculation using ABC/2 and provided with an electronic instruction manual. CT scans will be performed as standard procedures, and additional magnetic resonance imaging or other scans will be used when patients appear to have neurological deterioration. The final results of the three CT scans in every patient will be evaluated by independent investigators blinded to the clinical variables using the 3D Slicer software package (version 4.10.2; National Institutes of Health, Bethesda, MD, USA). ICH and PHE volumes will be measured using a semiautomatic volumetric algorithm as in a previous study (Figure 2) (22, 23).

#### BG Monitoring

During the first 7 days after enrollment, BG will be monitored 3 times a day. In patients with hyperglycemia, BG will be monitored at least every 2 h until BG returns to normal.

#### Post-discharge Follow-Up

A telephone follow-up after discharge is planned at 90 days after enrollment. Post-discharge follow-up will be performed by the investigators in every center who did not participate in other parts of the trial, including the randomization or treatment of the subjects. When all efforts to follow-up have been made but have failed, the patients will be recorded as lost to follow-up at the end of the case report form.

### Study Outcomes

#### Efficacy Outcomes

The primary efficacy endpoint is the percentage of unfavorable outcomes (mRS ≥3) at 90 days post-enrollment. The secondary efficacy endpoints include changes in the volume of ICH and PHE between the baseline and follow-up CT scans, and changes in the clinical scores between baseline and follow-up.

#### Safety Outcomes

The safety endpoints include the BG-related safety and cardiac-related safety of glibenclamide tablets as follows: (1) hypoglycemia (BG <3.1 mmol/L), (2) symptomatic hypoglycemia (hypoglycemia with confirmed hypoglycemic symptoms), (3) incidence of cardiac AE/SAEs or a QT interval of >500 ms, and (4) incidence of all-cause mortality.

### Blinding

The outcome assessors and data analysts will be blinded to the interventions. There will be one investigator assessing the functional outcomes in every center who will be blinded to the intervention received by the participants. All of the imaging evaluations will be conducted by independent investigators blinded to the clinical variables, interventions, and functional outcomes. The analyses will be conducted by blinded biostatisticians.

### Data Quality

To narrow the gap of uniformity between the centers and ensure the quality of the study, the following guidelines will be implemented: (1) an initial meeting will be held for all of the principal investigators and research coordinators from the participating centers before the commencement of the GATE-ICH study; (2) in each research center, the principal investigator (PI) will administer the trial and the monitor the quality of the data; (3) to ensure the quality of the trial, training sessions, phase meetings, and monthly monitoring visits at every center will be required; and (4) the Quality Control and Assurance Committee will prepare monthly reports regarding the progress of the trial, including patient recruitment, randomization, protocol adherence, and completeness of the data. The informed consent forms, case report forms, reports of AE/SAE and CT data of Digital Imaging and Communications in Medicine (DICOM) will be securely stored in a locked file cabinet in a secure office. All of the digital data will be stored in a password- and firewall-protected secure computer. The sponsor of the trial will have access to the final data.

### Monitoring

The PI in each center will manage quality control by monitoring the administration of the trial in accordance with the protocol, applicable guidelines, and regulations. Monthly reports regarding the progress of the trial will be prepared by the Quality Control and Assurance Committee, including patient recruitment, randomization, protocol adherence, reports of AE/SAE and completeness of the data. All participating sites will have phase meetings and monthly monitoring visits to verify consent, eligibility criteria, anomalous data, and reported SAEs.

### Determination of the Sample Size

The sample size was set at 220 to provide at least 80% power (1-β) to detect an 23.3% absolute risk reduction in the primary efficacy outcome for patients in the glibenclamide group compared to those in the control group, using a test of two-sided significance with a 5% type I error (α). The rate of non-adherence to the treatment protocol and overall loss to follow-up is assumed to be 15%. In our pilot study, the volume of PHE in patients receiving glibenclamide was significantly smaller than control group at day 7 (21.74 ± 9.70 mL vs. 31.22 ± 10.16 mL, *p* = 0.038). At 90 days, 33.3% of patients in control group had unfavorable outcomes while the data was 10.0% for the patients assigned to glibenclamide group (Supplementary File 2).

### Statistical Considerations

Patients in our study will be analyzed according to the intention-to-treat principle. Analyses will be conducted by blinded biostatisticians. Baseline data will be analyzed using univariate analyses. Categorical variables will be presented as rates, whereas continuous variables will be expressed as median (interquartile range) or mean ± standard deviation. The primary and secondary outcomes will be compared between patients randomized to the glibenclamide group and the control group using the Chi-square test or Fisher's exact-test and the Student's *t*-test or Wilcoxon rank-sum-test. Logistic multivariate analyses will be used to adjust for potential confounding effects of different variables and estimate the adjusted odds ratios and associated 95% confidence intervals. Two-sided *p*-values < 0.05 in all tests will be considered significant. The statistical analysis will be performed with SPSS version 19.0 software (SPSS Inc., Chicago, IL, USA).

### Patient and Public Involvement

Patients were not involved in the design of the study. The burden of intervention and the participation time in the research will not be assessed by the patients themselves. During the treatment stage, analysis of adverse reactions (such as symptoms of low blood sugar) will require timely feedback from the patients, assessment of the investigators, and confirmation by assessment instruments. At the termination of the trial, a scientific article will be authored to present the main results, and a brief summary of the results in plain language will be provided to all participants.

### Protocol Amendments

Any amendments to the protocol will be requested with the agreement of the GATE-ICH Study Group, Sponsor, and Funding Body. Following approval by the ethics committee, the modifications to the protocol will be communicated to the trial investigators and the trial registry (and if required, to the trial participants).

## Discussion

The GATE-ICH trial is a multicenter, randomized, controlled, assessor-blinded clinical trial in China. We will assess the hypothesis that small doses of oral glibenclamide is an effective method to reduce the PHE after ICH and to improve the 90-day prognosis of patients. The trial will provide new evidence regarding the management of PHE in patients with ICH.

In patients with supratentorial ICH, the hematoma volume was one of the important factors predicting outcomes (1). Patients with massive ICH (volume >30 mL) were associated with an increased risk of poor outcomes, and might respond better to surgical treatment rather than conservative treatment (24, 25). In addition, the clinical outcomes were not influenced by the PHE in patients with hematoma volume >30 mL no matter the location (10, 26). Results of previous studies found that the effect of PHE on 90-day unfavorable outcome was more relevant in patients with hematoma volume <30 mL than those with massive ICH (26–29). In patients with hematoma volume <30 mL, there was a significant association between PHE and poor outcome in basal ganglia ICH (10, 26). However, the relationship of PHE and poor outcome was controversial in lobar ICH (10, 26). Thus, basal ganglia hematomas with volumes <30 mL could be regard as an optimal target for interventions designed to ameliorate PHE after ICH (10).

Although the conservative treatment was usually suggested in patients with hematoma volume <30 mL, there are few effective therapies available for treatment of PHE after ICH (25). Traditional dehydration therapy is the most common option in clinical practice; however, its' effect is modest (7, 30). Identifying anti-edema drugs may be a promising intervention for ICH. As a candidate anti-edema drug, glibenclamide has been proven effective for the treatment of brain swelling after large hemispheric infarction in humans (12). In the GAMES-Pilot study, vasogenic edema appears to be reduced in patients with acute ischemic stroke treated with glibenclamide (16). It has been verified that glibenclamide could improve the injury of ICH through its' anti-inflammatory effects and scavenging of free radicals in pre-clinical studies (14, 15). Our study aims to test the efficacy of glibenclamide for treating edema and improving 90-day outcomes after ICH in a treated group compared to a control group in human subjects.

The GATE-ICH study is a multicenter randomized, controlled, assessor-blinded trial. Some limitations of this trial should also be noted. First, as a definite limitation in this trial, the placebo control was originally designed at the beginning of the design phase. However, unexpected failure of the placebo production line forced us to adjust initial study protocol from the placebo control to the blank control. After rigorous evaluation, the main outcome was a subjective outcome indicator and might be biased by the placebo effect, while both of imaging outcomes and safety outcomes were objective outcomes with limited placebo effect. In order to avoid the influence of placebo effect as much as possible, the outcomes evaluation and statistical analysis would be blindly processed by independent researchers throughout the study. Second, some background treatments were not standardized. For example, the selection of osmotherapy medicines depended on local availability in patients with intracranial hypertension. Thus, background treatments should be standardized as much as possible in future studies. Last but not least, the population was limited to the Chinese patients, which might restrain generalizability of the results to other populations. Thus, multicenter research in more countries will be needed.

### Trial Status

We conceived and designed the present trial in 2018. The trial was registered on November 8, 2018 (ClinicalTrials.gov, NCT03741530) and initiated on November 25, 2018. The protocol was version 5 on May 6, 2019. At the time this manuscript is being submitted, the enrollment and follow-up of patients is currently ongoing. This trial will be end in the second quarter of 2021.

## Ethics Statement

The studies involving human participants were reviewed and approved by The Ethics Committee of Xijing Hospital. The patients/participants provided their written informed consent to participate in this study.

## Author Contributions

This trial was conceived by FY and WJ. WJ is the chief investigator. JZ is a sub-investigator. CS assists with the study and analysis. LL, XY, XW, LY, JG, KW, and FF are the principal investigators and consulting clinicians at each study site. The first version of the manuscript was produced by JZ and FY with advice from WJ. All authors are responsible for the designation and conduction of this study as well as the data analysis, the drafting, and the reviewing of the final content of the paper.

## Conflict of Interest

The authors declare that the research was conducted in the absence of any commercial or financial relationships that could be construed as a potential conflict of interest.
