# Supplementary material for: Glibenclamide Advantage in Treating Edema After Intracerebral Hemorrhage (GATE-ICH): Study Protocol for a Multicenter Randomized, Controlled, Assessor-Blinded Trial
Source: Front Neurol. 2021 Apr 27;12:656520. doi: 10.3389/fneur.2021.656520 (PMC8110908; doi:10.3389/fneur.2021.656520)
Supplement: Supplementary file 2 [file Data_Sheet_2.docx]

**Supplementary file 2**

1. **Pilot study**
   1. **Patients**

During the enrollment, a total of 52 patients with ICH were screened. According to the inclusion criteria and exclusion criteria, 22 patients with ICH were included in the final analysis of pilot study. 10 patients received glibenclamide tablets and usual background care, while the other 12 patients received usual background care only.


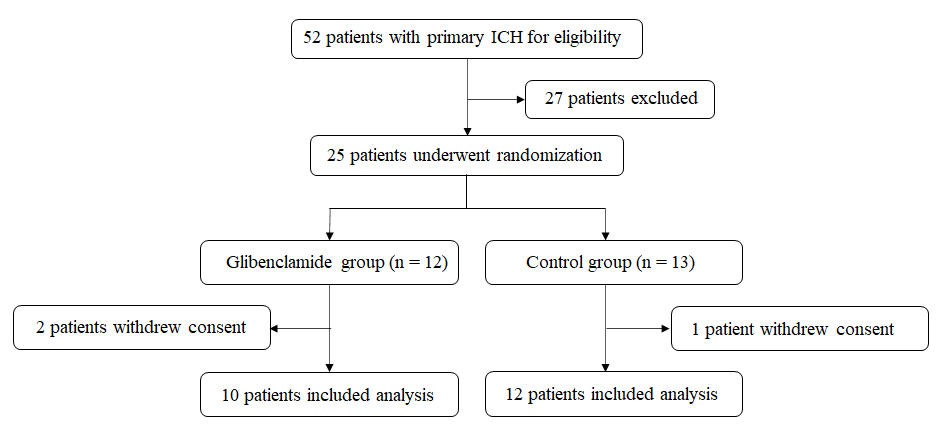


**1.2 Blood glucose**

At the first 4 hours after enrollment, the mean blood glucose was 7.52 mmol/L (8.2% reduced from baseline) in the glibenclamide group as compared with 11.31 mmol/L (58.0% increased from baseline) in the control group (P = 0.046). The glibenclamide group showed stable mean blood glucose with range from 6.4 to 9.6 mmol/L, while the control group showed intermittent elevated blood glucose with range from 7.2 to 12 mmol/L.


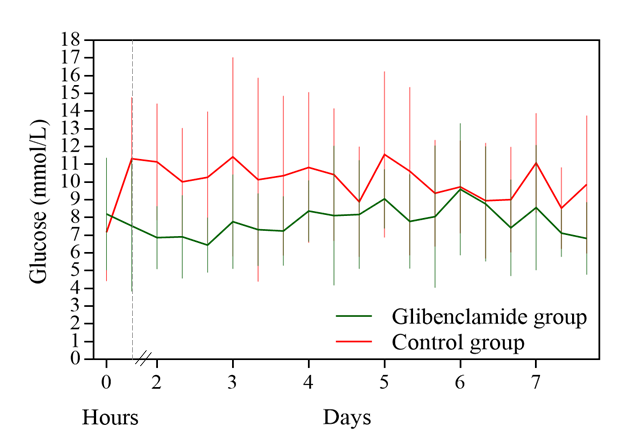


**1.3 Imaging evaluation**

The intraclass correlation coefficient between two independent researchers was 0.992 (p < 0.001). The mean (±SD) decreases in ICH volume (change from baseline to day 7) was more in glibenclamide group than in control group (3.26 ± 2.35 vs. 1.27 ± 2.11, p = 0.050, Figure a). Patients in control group showed larger expansion of PHE volume than those in glibenclamide group (PHE increase from baseline to day 7: 19.19 ± 7.49 mL vs. 8.80 ± 7.65, p = 0.004, Figure b). The rPHE showed similarly enlargement in both groups (Figure c).


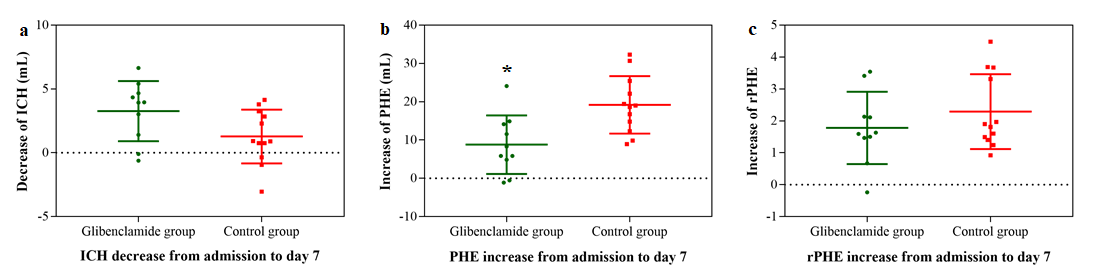


**1.4 Clinical Outcomes**

The baseline median clinical scores (NIHSS, GCS, mRS, Barthel index) were similar in both groups. There was also no significant difference in median clinical scores at discharge between glibenclamide group and control group. However, patients treated with glibenclamide had significantly increase of Barthel index from baseline to discharge compared with control group (20.0 [10.0-21.3] vs. 5.0 [0.0-15.0], p = 0.017). At day 90, the Barthel index was significantly higher in patients treated with glibenclamide than the control group. The decrease of mRS from baseline to day 90 was significant in glibenclamide group than control group (3.0 [2.0-3.0] vs. 2.0 [1.0-2.0], p = 0.007). The overall benefits of glibenclamide included both short-term and long-term clinical recovery.

**Table S1 Clinical outcomes of the participants**

|  | **Glibenclamide group (n = 10)** | **Control group**  **(n = 12)** | **P value** |
| --- | --- | --- | --- |
| NIHSS |  |  |  |
| Baseline | 13.0 (6.8-20.0) | 11.0 (7.3-24.5) | 0.741 |
| Discharge | 8.0 (4.3-11.3) | 8.0 (3.0-12.8) | 0.716 |
| NIHSS decrease from baseline to discharge | 3.5 (1.8-10.3) | 1.5 (0.0-6.0) | 0.320 |
| GCS |  |  |  |
| Baseline | 14.0 (10.5-15.0) | 13.5 (10.5-15.0) | 0.973 |
| Discharge | 15.0 (14.0-15.0) | 15.0 (14.3-15.0) | 0.660 |
| GCS increase from baseline to discharge | 1.0 (0.0-4.3) | 0.5 (0.0-2.8) | 0.728 |
| mRS |  |  |  |
| Baseline | 4.0 (3.8-5.0) | 4.0 (4.0-4.8) | 0.590 |
| Discharge | 4.0 (3.5-4.0) | 4.0 (3.0-4.0) | 0.941 |
| Day 90 | 1.5 (0.8-2.0) | 2.0 (2.0-3.0) | 0.065 |
| mRS decrease from baseline to discharge | 0.5 (0.0-1.0) | 0.0 (0.0-0.8) | 0.182 |
| mRS decrease from baseline to day 90 | 3.0 (2.0-3.0) | 2.0 (1.0-2.0) | 0.007 |
| mRS 0-1 on day 90 (N, %) | 5 (50.0) | 2 (16.7) | 0.172 |
| mRS ≥ 3 on day 90 (N, %) | 1 (10.0) | 4 (33.3) | 0.323 |
| Barthel index |  |  |  |
| Baseline | 5.0 (0.0-45.0) | 15.0 (10.0-30.0) | 0.457 |
| Discharge | 25.0 (20.0-58.8) | 25.0 (10.0-71.3) | 0.481 |
| Day 90 | 90.0 (83.8-100.0) | 80.0 (63.8-83.8) | 0.016 |
| Barthel index increase from baseline to discharge | 20.0 (10.0-21.3) | 5.0 (0.0-13.8) | 0.017 |
| Barthel index increase from baseline to day 90 | 62.5 (47.5-91.3) | 52.5 (10.0-68.8) | 0.145 |
| Barthel index 95-100 on day 90 (N, %) | 1 (8.3) | 4 (40.0) | 0.135 |

GCS, Glasgow Coma Scale; mRS, modified Rankin Scale; NIHSS, National Institutes of Health Stroke Scale.
